# Chronic Oral Pelargonidin Alleviates Learning and Memory Disturbances in Streptozotocin Diabetic Rats

**Published:** 2011

**Authors:** Mohammadali Mirshekar, Mehrdad Roghani, Mohsen Khalili, Tourandokht Baluchnejadmojarad

**Affiliations:** a *Department of Physiology, School of Basic Sciences, Shahed University, Tehran, Iran.*; b *Department of Physiology, School of Medicine, Shahed University and Medicinal Plant Research Center, Tehran, Iran.*; c *Department of Physiology, School of Medicine, Tehran University of Medical Sciences, Tehran, Iran.*

**Keywords:** Pelargonidin, Learning and memory, Spatial memory, Cognition, Diabetic rat, Oral Pelargonidin

## Abstract

Diabetes mellitus is accompanied with disturbances in learning, memory, and cognitive skills in the humans and experimental animals. Due to the anti-diabetic and antioxidant activity of pelargonidin (PG), this research study was conducted to evaluate the efficacy of chronic oral PG on alleviating learning and memory disturbance in streptozotocin-diabetic rats. Male Wistar rats were divided into control, diabetic, PG-treated control and PG (single-and/or multiple-dose)-treated diabetic groups. PG was administered p.o. once at a dose of 10 mg/kg and/or multiple doses on alternate days for 8 weeks. For induction of diabetes, streptozotocin (STZ) was injected IP in a single dose of 60 mg/kg. For the evaluation of learning and memory, initial latency (IL) and step-through latency (STL) were determined at the end of study using a passive avoidance test. Meanwhile, spatial memory was assessed in a Y-maze task. It was found that the alternation score of the diabetic rats was lower than the control (p < 0.05) and that single dose PG-treated diabetic rats (p < 0.05) showed a higher alternation score in comparison with the diabetic group. Regarding initial latency, there was no significant difference among the groups. In addition, diabetic and single-dose PG-treated diabetic rats developed a significant impairment in retention and recall in the passive avoidance test (p < 0.01), as was evident by a lower STL. Furthermore, the retention and recall of multiple-dose PG-treated diabetic rats was significantly higher in comparison with diabetic rats (p < 0.05). Therefore, it can be concluded that single-dose oral PG may attenuate spatial memory in the Y maze paradigm and multiple-dose chronic PG could improve retention and recall capability in the passive avoidance test in STZ-diabetic rats.

## Introduction

Diabetes mellitus (DM) is a common metabolic disorder characterized by hyperglycemia due to an absolute or relative insulin deficiency (1). DM is known to be associated with neurological complications in both the peripheral (PNS) and central (CNS) nervous system (2). Regarding PNS, DM leads to a wide range of peripheral neuronal deficiencies including reduced motor nerve conduction velocity, impaired sciatic nerve regeneration, axonal shrinkage in association with reduced neurofilament delivery, and deficient anterograde axonal transport (3). In rats with diabetes experimentally induced by streptozotocin (STZ), the nerve damage observed parallels in many ways with the nerve degeneration seen in human diabetic neuropathy (4). In addition, different kinds of neuropathies are one of the major complications contributing to morbidity in patients with diabetes mellitus. Recently, pathological studies have suggested that diabetes is one of the risk factors for senile Alzheimer’s dementia (5). Although many studies regarding the relationship between diabetes and peripheral neuropathy have carried out to date, yet, the effects and consequences of diabetes on the brain itself have not been studied much. To date no marked structural abnormality has been found in the central nervous system of patients with diabetic neuropathy using routine histochemical staining methods (6). Until now, very few etiological studies on the relationship between diabetes, learning and memory have been conducted. Manifestations of cerebral disorders in diabetic patients include alternations in neurotransmission, electrophysiological abnormalities, structural changes and cognitive deficits (7). In this regard, it has been reported that diabetic patients have brain atrophies, a two-fold increased risk of dementia, and impaired learning and memory. In addition, diabetic rats exhibit variant degrees of spatial learning impairments (8). It is a well-known fact that a cognitive deficit is associated with changes in hippocampal synaptic plasticity including an impaired expression of long-term potentiation (LTP) and an enhanced expression of long-term depression (LTD) (8-9). From a functional viewpoint, DM is reported to specifically impair memory function in experimental animals with strong involvement of the hippocampus and cerebral cortex. Interactions between glucose and cognitive functions have been reported both in the presence of elevated arterial blood glucose levels and with decreased cerebral glucose metabolism. These findings may indicate a disturbed acquisition and/or consolidation of memory (10).

On the other hand, recent research has been focusing on the use of non-vitamin antioxidants such as flavonoids to reduce the devastating complications of diabetes in experimental animals and patients (11). Plant-based pharmaceuticals including flavonoids have been employed to control various diseases afflicting mankind (11). Flavonoids are an essential part of the human diet and are present in plant extracts that have been used for centuries in oriental medicine. The antioxidant properties, ROS scavenging, and cell function modulation of flavonoids could account for a large part of their pharmacological activity (11, 12). Since diabetes mellitus is considered as being a free radical-mediated disease, there has been renewed interest in the use of flavonoids in diabetes research. Anthocyanins and their aglycone derivatives anthocyanidins are important groups of flavonoids. Of these groups, pelargonodin has been reported to exhibit antidiabetic activity in diabetic rats (13, 14). Recent reports have also demonstrated multiple benefits associated with the consumption of pelargonidin-rich fruits including decreased vulnerability to oxidative stress, reduced ischemic brain damage, protection of neurons from stroke-induced damage and the reversal of age-related changes in brain behavior (15). Berry fruits contain high amounts of anthocyanins, which play a major role as free radical scavengers and could inhibit H_2_O_2_-induced lipid peroxidation in the rat brain homogenates (16). Therefore, this study was undertaken to evaluate the efficacy of chronic oral pelargonidin on alleviation of learning and memory disturbance in streptozotocin-diabetic rats using passive avoidance and Y-maze paradigms.

## Experimental

*Animals*

Male albino Wistar rats (Pasteur’s institute, Tehran, Iran) weighing 215-285 g (10-12 weeks old) were housed in an air-conditioned colony room on a light/dark cycle (21-23°C and 30-40% humidity) and supplied with a standard pelletized diet and tap water *ad libitum*. Procedures involving animals and their care were conducted in conformation with the NIH guidelines set out for the care and use of laboratory animals.

The rats (n = 40) were randomly allocated and similarly grouped into five groups: normal vehicle-treated control, PG-treated control (multiple dose), vehicle-treated diabetic, PG-treated diabetics (single dose), and PG-treated diabetic (multiple dose). The rats were rendered diabetic by a single intraperitoneal injection of 60 mg kg^-1^ STZ freshly dissolved in normal cold saline. Control animals received an injection of an equivalent volume of normal saline and vehicle. Diabetes was confirmed by the presence of hyperglycemia, polyphagia, polydipsia, polyuria and weight loss. One week after the STZ injection, blood samples were collected and serum glucose concentrations were measured using the glucose oxidation method (Zistshimi, Tehran). Only those animals with serum glucose levels higher than 250 mg dL^-1^ were judged as being diabetic and deemed suitable for use in the following experiments. The day on which hyperglycemia was confirmed was designated as day 0. PG was administered p.o. (using a gavage needle) at a dosage of 10 mg/kg body weight one time and/or on alternate days one week after STZ injection for a period of 8 weeks. PG was dissolved in 10% cremophor with further dilution in distilled water before use. Changes in body weight, food consumption and water intake were regularly observed during the experimental period. Behavioral tests including passive avoidance and Y-maze were performed at the end of study in experimental groups as described below.

*Y-maze task*

Short-term spatial memory performance was assessed by recording spontaneous alternation behavior in a single Y-maze session (6). The maze was made up of black-painted Plexiglas. Each arm was 40 cm long, 30 cm high and 15 cm wide. The arm converged in an equilateral triangular central area that was 15 cm at its longest axis. The procedure was basically the same as that described previously as follows: each rat, naive to the maze, was placed at the end of one arm and allowed to move freely through the maze during an 8 min session. The series of arm entries were recorded visually. Arm entry was considered to have been completed when the base of the animal’s tail was entirely placed in within the arm. Alternation was defined as successive entries into the three arms on overlapping triple sets. For instance, each alternation is followed by a comma in the following sequence of arm entries (each arm is labeled A, B, or C): ACBCACACBACABCA. In this example, the rat entered 15 arms, 9 of which are alternations. The number of maximum spontaneous alternations is therfore the total number of arms entered subtracting 2 and the percentage is calculated as the ratio of actual to possible alternations (defined as the total number of arm entries minus two). In this instance, the alternation percentage will be 69.2%.

*Single-trial passive avoidance test*

This test was conducted 2-3 days after the Y-maze task. The apparatus (BPT Co., Tehran) consisted of an illuminated chamber connected to a dark chamber by a guillotine door. Electric shocks were delivered to the grid floor via an isolated stimulator. On the first and second days of testing, each rat was placed on the apparatus and left for 5 min to habituate to the apparatus. On the third day, an acquisition trial was performed. Rats were individually placed in the illuminated chamber. After a habituation period (5 min), the guillotine door was opened and after the rat entered the dark chamber the door was closed. An inescapable scrambled electric shock (1 mA, 1 sec once) was then delivered. In this trial, the initial latency (IL) of entrance into the dark chamber was recorded and rats with ILs of greater than 60 sec were excluded from the study. Twenty-four h later, each rat was placed in the illuminated chamber for a retention trial. The interval between the placement in the illuminated chamber and the entry into the dark chamber was measured as step-through latency (STL up to a maximum of 480 sec as cut-off).

*Statistical analysis*

All results were expressed as mean ± SEM. For the behavioral tests including the passive avoidance test and the Y-maze task, non-parametric Kruskal-Wallis and Mann-Whitney rank sum tests were used. Body weight and serum glucose levels at various weeks were analyzed using repetitions of the one-way ANOVA test. In all calculations, a difference of p < 0.05 was regarded as being significant. 

## Results

*General considerations*

**Figure 1:**
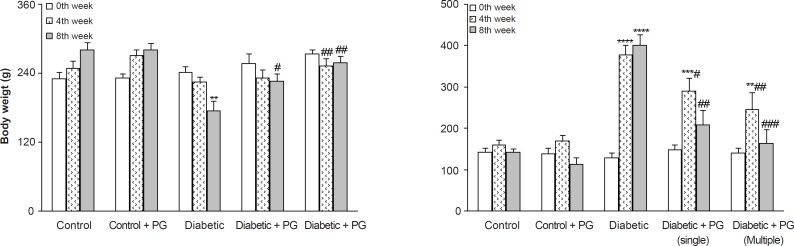
Body weight and serum glucose concentration in various weeks (means ± SEM). PG stands for pelargonidin. ** p < 0.01, *** p < 0.005, **** p < 0.0001 (as compared to week 0 in the same group); # p < 0.05, ## p < 0.01, ### p < 0.005 (as compared to diabetics in the same week)

After 8 weeks, the weight of the vehicle-treated diabetic rats was found to have significantly decreased in comparison to the control rats (p < 0.01) and PG treatment (single and multiple doses) caused a less significant decrease in diabetic rats as compared to non-diabetics (p < 0.05-0.01). In this respect, PG administration for 8 weeks (multiple doses) was more effective in preventing weight loss than a singular dose. In addition, diabetic rats also had an elevated serum glucose level than those of control rats (p < 0.001) and treatment of diabetic rats with PG (single and multiple doses) caused a significant decrease in their serum glucose (p < 0.01-0.005) relative to vehicle-treated diabetics. Again, PG administration for 8 weeks was more effective in reducing serum glucose than a singular dose. Meanwhile, PG treatment in control rats did not produce any significant change with regards to serum glucose levels (Figure 1). 

*Alternation behavior in Y-maze*

**Figure 2:**
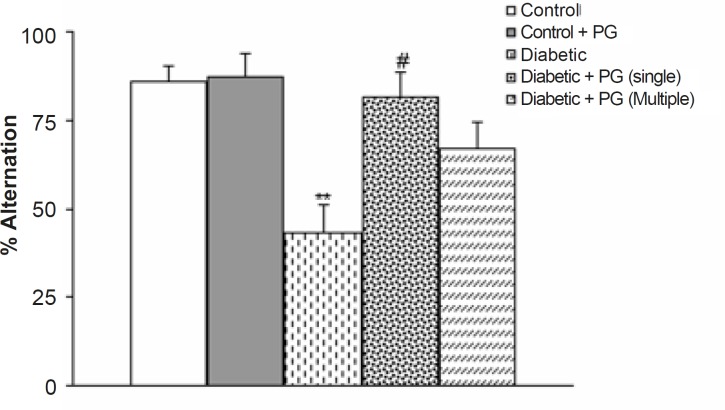
Alternation behavior of treated-control and -diabetic rats in Y-maze task. PG stands for pelargonidin.** p < 0.01 (as compared to control group), # p < 0.05 (as compared to diabetic group).

Figure 2 shows the results of the Y-maze task, in which short-term spatial memory performance can be examined. There was no significant difference in the total number of times the animal entered an arm. However, the alternation score of the diabetic rats was lower than that of the control ones at the end of 8 weeks (p < 0.05). Meanwhile, PG (single dose)-treated diabetic rats showed a higher alternation score in comparison with the untreated diabetic group (p < 0.05) at the end of the study. Meanwhile, PG treatment of control rats did not produce any significant change regarding this parameter. 

**Figure 3:**
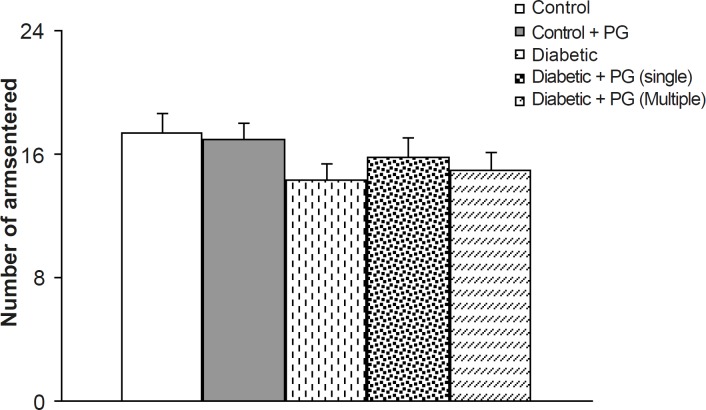
Number of arms entered in Y-maze task in different groups. PG stands for pelargonidin

To avoid the compounding effect of locomotor activity on memory processes in experimental groups, especially diabetics, the total number of arms entered was considered as an index of locomotion. In this respect, although the total number of entrances was less in diabetic animals, especially in untreated diabetic rats, this difference was not statistically significant when compared to the controls. In addition, there were also no significant differences among the groups (Figure 3).

*Passive avoidance test*

**Figure 4:**
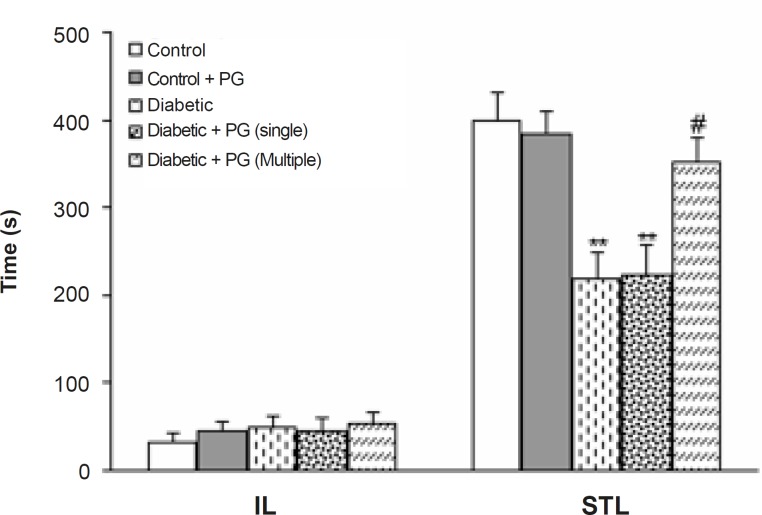
Initial (IL) and step-through (STL) latencies of treated-control and -diabetic rats in single-trial passive avoidance test.** p < 0.01 (vs. control group), # p < 0.05 (vs. diabetic group

Figure 4 shows the performance of treated-control and diabetic rats in the passive avoidance paradigm as indicated by initial and step-through latencies. Regarding initial latency, there was no significant difference among the groups. In addition, diabetic and single dose PG-treated rats developed a significant impairment in retention and recall in the passive avoidance test (p < 0.01), as is evident by a lower STL. Furthermore, the retention and recall of multiple-dose PG-treated diabetics was higher in comparison with diabetic rats (p < 0.01). Meanwhile, PG treatment of control rats did not produce any significant change in this regard.

## Discussion

The aim of this study was to determine whether chronic oral pelargonidin alleviates learning and memory disturbance in streptozotocin-diabetic rats. The results clearly demonstrated that long-term diabetes is accompanied with disturbances in animal performance in the passive avoidance and Y-maze task. In addition the results show that single-dose pelargonidin improves spatial memory performance and its administration in multiple doses could prevent retention and recall abnormality in diabetic rats. 

Previous studies have reported that DM is associated with neurological complications in both the PNS and CNS. The impairment of learning and memory is also recognized as being a complication of diabetes (17). Cognitive deficits in DM can result from metabolic impairment or cerebral vascular complications (1). In animal models of DM, spatial learning impairments have also been reported. STZ-diabetic rats also display deficits in cognitive tasks, such as performance in the Morris water maze (4). Although the pathogenesis of these deficits is multi-factorial and controversial, there is strong evidence for the involvement of microvascular dysfunction and oxidative stress due to an excessive production of oxygen free radicals. In the latter case, as the mammalian hippocampus and cerebral cortex play a pivotal role in a diverse set of cognitive functions, such as novelty detection and memory, these areas are very vulnerable to oxidative damage in STZ-diabetic animals (18). In agreement with this idea, it has been reported that lipid peroxidation enhances in both regions of the brain, which in itself leads to a significant impairment in both motor and behavioral memory functions in diabetic animals (9). On the other hand, previous studies in rats have found that an induction of diabetes impairs long-term potentiation (LTP) and enhances long-term depression (LTD) induced by high frequency (HFS) and low frequency stimulations (LFS) respectively. This could indicate that diabetes acts on synaptic plasticity through mechanisms involved in metaplasticity. It is also known that LTP plays a crucial role in the consolidation of memory. In the present study, diabetic rats also showed learning impairment in the Y-maze task, which is an indicator for spontaneous alternation behavior. As was shown in Figure 4, the score of alternation behavior in diabetic rats was significantly lower than that of the control. Other tasks aimed at estimating the learning abilities of diabetic rats have previously been attempted. Such tasks request certain types of motivation, *e.g.*, food, water, or swimming (6). Body weight, the intake of food or water, and the spontaneous motor activity of diabetic rats were significantly different from those of control rats. The Y-maze task is a moderate task that does not require such motivations. Our finding that the alternation score of diabetic rats was lower than that of control ones, is evidence of memory impairment in a diabetic animal model (19). 

The beneficial effect of PG in this study may be attributed partly to its anti-hyperglycemic effect and to some extent to its attenuation of free-radical-mediated lipid peroxidation in those brain regions critical for animal performance in Y-maze and passive avoidance tests. Literature reports increased lipid peroxidation products and remarkable changes in free radical scavenger system enzymes in diabetic patients and different organs from diabetic animals (20). In addition, cognitive dysfunction due to experimental diabetes could be attributed in some extent to increased oxidative stress in related brain centers (21). Increased intracellular sorbitol has also been associated with cellular tissue damage and it is believed that the damage is due to either the increase in intracellular osmolality or the reduction of the cellular redox state, making the cells more susceptible to oxidative damage (22). Increased intracellular sorbitol is associated with a reduction of intracellular taurine, a common osmole regulator and antioxidant (22). Taurine has also been shown to be an important neurotrophic factor in the retina and brain (22). Increased activity of the polyol pathway in the peripheral nerve is associated with a reduction of intracellular inositol, a major component of phospholipids and the production of neurotransmitters (22). Moreover, increased activity of the polyol pathway has been reported to reduce the production of nerve growth factor from Schwann cells, which could also be a mechanism for reduced neuronal growth (22). These observations indicate mechanisms for brain injury associated with hyperglycemia (22). In our study, as PG administration exhibited a hypoglycemic effect, this could alleviate some of the above-mentioned abnormalities in the brain of diabetic animals. 

In our study, contrary to our expectation, the performance of rats in the Y-maze test as evaluated by alternation behavior was superior in single-dose PG-treated diabetic rats than multiple-dose PG-treated diabetic ones. In addition each of both of these groups performed better in comparison to untreated diabetic rats. This could be attributed to the extent of stress felt by the animals receiving treatments through a gavage needle. It has been reported that chronic stress, as may be the case for multiple-dose PG, may improperly affect the cholinergic pathway originating from the cerebral cortex and in this way disturb some aspects of spatial memory (23, 24) 

In conclusion, single-dose oral PG could attenuate the spatial memory in the Y-maze paradigm, and multiple-dose chronic PG could improve the retention and recall capability of STZ-diabetic rats in passive avoidance tests. Further studies are warranted to investigate the involved mechanisms in further detail. 
